# Comprehensive Neurosurgery Board Review

**DOI:** 10.4103/2152-7806.74149

**Published:** 2010-12-22

**Authors:** Pieter L. Kubben

**Affiliations:** Department of Neurosurgery, Maastricht University Medical Center, The Netherlands


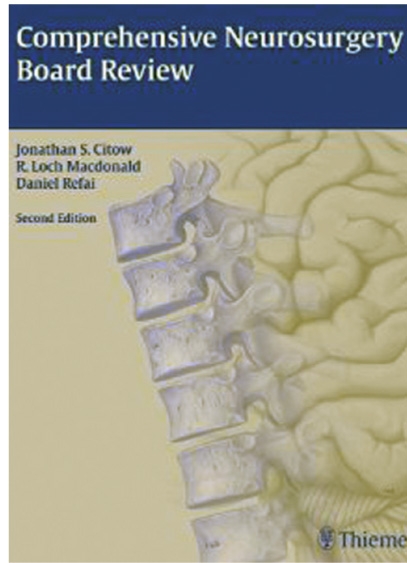


## DESCRIPTION

The second edition of this book is edited by Jonathan S. Citow, R. Loch Macdonald and Daniel Refai. It is published by Thieme Medical Publishers, Inc (New York) in 2010. This soft cover edition on A4 format consists of circa 530 pages content, and a list of abbreviations plus the index at the end.

## FIRST IMPRESSION

Good. The book offers clear illustrations in color and monochrome, the font is readable, and I am especially pleased to see not only CT and MR images, but also microscopic images. Although I personally prefer a smaller “handheld” format for studying, I am pleased with the book. It has clear tables, and seems to cover the important topic in the six chapters it offers: Anatomy, Physiology, Pathology and Radiology, Neurology, Neurosurgery, and Critical Care.

## FURTHER EXPLORATION

The book does offer what it promises: a review of board exam questions. Disclaimer: I am not a U.S. resident, so I have not seen the U.S. board questions. Anyway, the book has highly-relevant content in a readable and well-illustrated context. That having been said, this is not a “reading book.” It is very concise, like Greenberg’s Handbook of Neurosurgery. The difference is not only the size (Greenberg is smaller, but has also a smaller font size making it more difficult to read), but also the presentation of the content. The “Greenberg” is more the book that tells you “what to do,” while the Board Review tells you “what to know” (in a very practical manner, by the way).

## DISCUSSION

The question is whether this book is a useful addition in the collection of neurosurgical literature that is already available. My answer to that question is “yes,” and I think this is demonstrated by the existence of a second edition. The authors have done a great job of collecting so much information and offering it in a readable format. The only drawbacks that I found are related to some formatting aspects. First, for a book of this kind, I would personally prefer a smaller size that makes it an easy-to-take travel companion for studying on-the-go, which is perfectly possible with the concise texts that it offers. Second, I think the text formatting may benefit from some color. Although I perfectly understand that full color would be too expensive, a two-color (or three-color) format with colored subheadings may make the design more attractive and may enhance the learing effect by offering more visual cues. As I am convinced that this type of book will need a third edition in a few years, these considerations may be worth thinking about. An overview of the most important positive aspects of the book and these suggestions for improvement are provided in Table 1.

**Table 1:** Overview of positive aspects and suggestions for improvement

Positive	Suggestions for improvement
Concise	More color in text layout
Useful illustrations and tables	Smaller book size
Covers all important fields	

## CONCLUSION

Comprehensive Neurosurgery Board Review is a good and concise review book on board exam questions, that deserves its place in the collection of neurosurgical literature. The text is readable, and is enhanced with useful illustrations. Although a little more color in the text and maybe a smaller overall size may improve the book even more, Citow and his colleagues should be congratulated for their excellent work, which will support many of our (future) colleagues.

